# Commercial sex work among university students: a case study of four public universities in Ghana

**DOI:** 10.1186/s12905-021-01251-2

**Published:** 2021-03-10

**Authors:** Fred Yao Gbagbo, Josephine Akosua Gbagbo

**Affiliations:** 1 Department of Health Administration and Education, Faculty of Science Education, University of Education Winneba, P.O. Box 25, Winneba, Ghana; 2 Department of Political Science Education, Faculty of Social Science, University of Education Winneba, P.O. Box 25, Winneba, Ghana

**Keywords:** Commercial-sex-workers, Ghana, Students, University

## Abstract

**Background:**

Despite its criminalization in Ghana, commercial sex work dates back to ancient societies and occurs in various forms within communities. The authors examined commercial sex work in selected public Universities in Ghana to inform policy and program decisions for safer sex at the universities in Ghana.

**Methods:**

The study was an exploratory-mixed-method design. Respondents were identified using purposive and snowballing techniques while semi-structured questionnaires and in-depth interviews were used for data collection between 2017and 2019. Quantitative data were analyzed using Statistical Package for Social Sciences version 23 and qualitative data analyzed thematically.

**Results:**

Findings show that there is a proliferation of commercial sex work on university campuses in Ghana for financial, material, and emotional gains. Student sex workers have devised various strategies to combine academic work and sex work. Prospective customers are solicited by hanging out in drinking bars and nightclubs in and around university campuses at night and/or leaving contact details with pimps to be contacted for services. Brothels are also springing up in and around the university campuses in the form of movie houses and student sex workers convert their hostel rooms into brothels. Price negotiation is based on the environment, duration, the sex workers' perceived safety of the sexual act, customer's preferences for styles, and positions adopted for sex.

**Conclusion:**

There is a need for further studies in this area and a multi-sectoral approach for appropriate policy and program interventions to regulate the practice on campus.

**Supplementary Information:**

The online version contains supplementary material available at 10.1186/s12905-021-01251-2.

## Background

The word commercial sex work (CSW) is derived from the Latin word “Prostare” which means publicly selling oneself [1]. Historically, commercial sex work dates back to ancient societies and occurs throughout human history [2]. This practice is an age-long phenomenon recorded between 640–556 B.C. [3] and characterized by the provision of sexual favors for financial [4–6] and non-financial rewards [7]. Despite its immoral undertone, the sale of sexual services is gradually becoming legalized in many countries worldwide [8], and terminologies used to describe commercial sex worker have evolved over the years from paid sex workers, commercial sex workers and slay queens among others to evade the associated stigma [9].

In Ghana, commercial sex work is criminalized [10] and defined as the offering of a person’s body for paid sex [11]. Despite its criminalization, commercial sex work is gaining ground even among children in various forms particularly in big commercial towns [12]. Anecdotal evidence in recent times has also shown the proliferation of commercial sex work in and around public Universities in Ghana. There are reported cases of university students indulging in commercial sex work on university campuses and/or leaving their contacts in various drinking spots, hotels and guest houses to be contacted by customers.

Although various studies exist on commercial sex work globally and in Ghana [13–15], little is known about the practice in public Universities within a restricted country like Ghana. This study, therefore, examined commercial sex work in and around public universities in Ghana, looking at student involvement, reasons for the practice, customers, modus operandi and cost of services. The findings of this study will provide empirical evidence to inform policy and program decisions for safer sex practices among students within public universities in Ghana.

### Theoretical framework

The study is based on a social constructivist epistemology which holds the notion that multiple truths exist to explain a phenomenon [16]. The constructivist approach for this study posits that eliciting multiple views on a human-centered issue and discussing the differences within a specific context gives a better understanding of the situation [16]. In this study, the social constructivist approach required the researchers to establish a good rapport with the student sex workers, and all significant others associated with these students solicit their experiences on the research topic.

## Methods

### Study design

The study design was an exploratory, mystery client and case study using a mixed-method (qualitative and quantitative) approach of data collection. This design was used to give a better understanding of the research problem [17]. The quantitative component focused on respondents’ characteristics, pricing determinates, cost of providing commercial sex services, regular customers of student sex workers, meeting places, and form of remuneration. The qualitative component sought to elicit participants’ accounts of their reasons [18] and experiences [19] in commercial sex work on campus. With this approach, the authors were able to investigate commercializing sex work among university students objectively without adding self-impressions or understanding to the findings.

### Study setting

The study was conducted in Accra, Kumasi, Cape Coast, and Winneba targeting students from the Public Universities located in these settings who are engaged in commercial sex work in and around these universities. Administratively, Accra is the capital of Ghana covering an area of 225.67 km^2^ (87.13 sq mi) with an estimated urban population of 4.2 million as of 2020. Kumasi is the second largest city of Ghana and the capital city of the Ashanti region, a very important and historical center for Ghana. Kumasi is located near Lake Bosumtwi, in a rainforest region, and is the commercial, industrial and cultural capital of Asanteman. Kumasi is approximately 480 km north of the Equator and 60 km north of the Gulf of Guinea. Kumasi is alternatively known as “The Garden City” because of its many beautiful species of flowers and plants. Cape Coast is a city, fishing port, and the capital of Cape Coast Metropolitan District and Central Region of south Ghana. It is also one of Ghana’s most historic cities known for its historical monuments of castles and forts used during the colonial era. Cape coast is also known for well-endowed secondary and tertiary academic institutions. The estimated population of cape coast is 143,015 as of 2020. Winneba, traditionally known as Simpa, is a town and the capital of Effutu Municipal District in the Central Region of South Ghana and is a historic fishing port in south Ghana, lying on the south coast, 140 km east of Cape Coast. Winneba has an estimated population of 55,331 as of 2020 [20]. The Universities in these settings were targeted because the authors perceived them as having the potentials for commercial sex work due to their location in a cosmopolitan area with high levels of commercial activities.

### Sampling

Twelve key informants (3 taxi drivers, 4 hostel attendants and, 5 receptionists at hotels/guest houses operating in and around the universities) were purposively selected, and tasked to help locate commercial sex workers, their locations and time of operations. Research assistants comprising two males, selected from each of the respective university campuses were trained for data collection, using self-administered structured questionnaires and semi-structured in-depth interviews. A total of 100 respondents comprising 25 students from each of the four public universities participated in the study. Twenty in-depth interviews were also conducted to further explore student’s experiences of combining commercial sex work and studies whilst on campus. The participants of the in-depth interviews were mainly randomly sampled final year student commercial sex workers (4 females from each of the universities) and all 4 male student commercial sex workers identified during data collection.

### Data collection procedure

The trained field assistants collected the data between 2017 and 2019 using a questionnaire and in-depth interview guides that were developed and pilot tested by the authors solely for this study. (An English language version has been uploaded as Additional file 1, Additional file 2 and Additional file 3). Respondents were selected purposively using snowball sampling techniques. A list of regular taxi drivers, hotels, and guest houses operating in and around the universities was made with the help of research assistants over a period of one month. Receptionists of the listed hotels and guest houses as well as the identified taxi drivers were then contacted one-on-one and informed about the nature, benefits, risks, purpose of the study, voluntary participation and, rights to withdraw. Those who consented to participate in the study were recruited and in-depth interviews [IDIs] were conducted with each of them at places deemed convenient and comfortable for them. The interviews focused on questions on student sex workers in and around the universities, availability of brothels/joint, time of operation, and how to contact the student sex workers. Information obtained from the key informants were used to identify the student sex workers recruited for the study. The in-depth Interview sessions lasted for 30 min equivalent to a ‘short time’ engagement of a commercial sex worker and were paid GHS 20 (US$ 1 would be equivalent to GHS 5.8) for their time as they would have charged a client. In some situations, extra money was paid to ensure data saturation since the commercial sex workers were concerned with time spent on engaging them. Data were collected in English and all interviews were handwritten with consent from respondents in the form of field notes.

## Data analysis

The quantitative data were analyzed using the Statistical Package for Social Sciences (SPSS) version 23 and presented in a frequency table whiles the qualitative data were analyzed thematically and results presented descriptively. All interviews were documented immediately and were read through to increase familiarity with the data. Data were analyzed using the thematic analysis approach. The documented responses were organized, coded, and managed manually. A list of code labels was created and a series of categories for the main themes that emerged were developed.

### Ethical considerations

Prior to the commencement of the study, the research protocol was presented at the bi-weekly academic research seminars of the Faculty of Science Education, University of Education, Winneba. The seminar brought together lectures of the Faculty (equivalent to an ethical review meeting) who critiqued and reviewed the study protocol for ethical suitability and sound methodology. The findings of the study were also subjected to the same scrutiny for endorsement before finalizing the report.

Verbal consents were obtained from all participants prior to data collection. Data were collected and managed in a way that did not compromise the privacy and confidentiality of anyone participating in the study. In protecting the privacy and confidentiality of both respondents and field assistants, details of their affiliated institutions, names, addresses, and contact numbers were excluded from the report. Additionally, there were no photographs of respondents nor recordings of the interviews which could be used to trace any field assistants and/or respondents. With these approaches, the findings of the study cannot be attributed to any particular individual, University, or particular places where the identified commercial sex workers operate. The field assistants were all oriented to behave professionally and none judgmental during the fieldwork.

## Results

### Background characteristics of respondents

In Table 1, the authors presented the background characteristics of the respondents. The majority of the respondent were young people aged 18–24 years (82%), Christians (78%), females(96%), unmarried (80%), foreign students (81%), being catered for by parents (52%), resides in an on-campus rented private hostel (62%) and undergraduates (79%) reading humanities (65%).

**Table 1:** Background characteristics of respondents. *Source*: Field data 2019

Variable	N	Percentage (%)
*Age in years*		
< 18	5	5.0
18–24	82	82.0
25–31	10	10.0
> 31	3	3.0
*Religion*		
Christian	78	78.0
Muslim	17	17.0
Other	5	5.0
*Sex*		
Males	4	4.0
Females	96	96.0
*Marital status*		
Married	13	13.0
Unmarried	80	80.0
Other	7	7.0
*Nationality*		
Ghanaian	19	19.0
Nigerian	63	63.0
Liberian	15	15.0
Other Nationals	3	3.0
*Primary care taker of respondents*		
Parents	58	58.0
Siblings	11	11.0
Partner	8	8.0
Self	17	17.0
Other	6	6.0
*Student residential status*		
On campus university hostel	62	12.0
On campus rented private hostel	23	62.0
Off campus rented apartments	3	23.0
Other	12	3.0
*Program of study*		
Humanities	65	65.0
Social Sciences	15	15.0
Health Sciences	4	4.0
Pure Sciences	10	10.0
Other	6	6.0
*Level of study*		
Certificate	12	12.0
Diploma	6	6.0
Undergraduate	79	79.0
Postgraduate	3	3.0

The following main themes under which the results are presented emerged during the thematic data analysis of the qualitative data:

1. Emergence of commercial sex work on public universities campuses
2. Mode of operations (modus operandi)
3. Respondents’ views about commercial sex work
4. Determinants of the cost of commercial sex workers' services on university campuses
5. Reasoning behind the prizing of commercial sex services
6. Initiation into commercial sex work
7. Reasons for commercial sex work
8. Regular customers of student sex workers
9. Meeting places for having sex
10. Form of remuneration for having sex
11. Challenges encountered by the student sex workers in the sex business

### The emergence of commercial sex work on public universities campuses

Available evidence from the key informants in this study shows that commercial sex work is being practiced on the campuses of the various public universities in various forms and students adopt various strategies and coping mechanisms for their sex business whilst in school. Students of various backgrounds including men were found as commercial sex workers for various reasons.

### Mode of operations (modus operandi)

It was reported that some of the student sex workers visit drinking bars and night clubs in and around the various university campuses at night in search of customers, whilst others operate in their hostel rooms or in a rented room (i.e. hotels, guest houses, and brothels) around the various university campuses. There are other categories that are highly paid female and male sex workers who work by appointment with an exclusive clientele. Such students leave their mobile phone contacts and pictures in hotels and guest houses in and around their respective universities to be contacted by prospective customers. Others also work with taxi drivers on the university campuses who link them up to customers, pick and drop them off at various locations at specified times for commissions. Brothels are also springing up in and around the university campuses in the form of movie houses and student commercial sex workers convert their hostel rooms into brothels. Some key informants reported that:

There are various joints, drinking bars and movie houses around this university that you can find student sex workers, particularly at night. I can take you there if you want to see for yourself (Taxi driver).

Another respondent indicated that:

We have contacts with some students who call us any time when they have a client to pick them for business. Sometimes we pick them to a hotel, guest house or the house of the customer and wait for them till they finish and we bring them back (Taxi driver).

Another respondent also revealed that:

Commercial sex workers are not allowed at this place but we have some students who come around to leave their contacts and pictures with us just in case a guest wants a woman to hang out with they can be contacted (Hotel receptionist).

### Respondents’ view about commercial sex workers

Generally, all respondents were of the view that commercial sex workers are unmarried women who have sex with men for material and financial gains. A respondent was of the view that:

If you are not married and having sex with men for any reason then technically you qualify as a commercial sex worker (Female, undergraduate student).

Another respondent indicated that:

Any woman who sleeps with more than one man for money is a commercial sex worker (Female undergraduate student).

### Determinants of the cost of commercial sex workers' services on university campuses

Commercial sex work among university students was observed as a business and the cost of their services was determined by place, duration, and type of services (Table 2) as well as the elegancy of the sexual position or styles adopted during the sexual act (Table 3).

**Table 2:** Cost of service by type, place and duration. *Source*: Field data 2019 (*US$ 1 would be equivalent to GH₵ 5.8*)

Type of service	Description	Place of service	Duration of service	Cost in GH₵
Short time	Sexual intercourse usually charged per ejaculation	Sex worker’s room	30minutes (1 round)	GH₵ 20–50
Sleep	An overnight sleep with sexual intercourse usually charged per 3 ejaculations	Sex worker’s room	Overnight (3 rounds)	Negotiable with a minimum of GH₵ 100–200
Away	Agencies and/or escort services such as taking Sex workers way from their environments on romantic dates and usually characterized with unlimited sexual intercourse	Any place outside the Sex worker’s room (e.g. Patron’s house, hotel rooms etc	2 days +	Negotiable with a minimum of GH₵ 500 per day

**Table 3:** Cost of service by sexual position/style as enumerated by respondents. *Source*: Field data 2019- (NB: These are all direct quotations from the respondents) (US$ 1 would be equivalent to GH₵ 5.8)

No	Sexual position/style	Description	Relevance	Cost *GH₵*
1	‘Missionary’	Lie on your back while he lies face down on top of you	This sex position is simple, relaxing, and less stressful to the woman	20
2	‘Face-off’	Your partner sits on a chair or the edge of the bed; you face him, seated on his lap	During this sex position, you’re in control of the angle and depth of the entry and thrust. Being seated adds support, so it’s great for marathon sex sessions	Negotiable
3	‘Doggy Style’	Get on all fours, then have your partner kneel behind you, with his upper body straight up or slightly draped over you (like a humping dog)	This sex position allows for deep penetration and easier G-spot stimulation	Negotiable
4	‘G-Whiz’	Lie back with your legs resting on each of your partner's shoulders	This sex position is awesome because when you raise your legs, it narrows the vagina and helps target the G-spot	Negotiable
5	‘Wheel barrow’	Get on your hands and feet and have him pick you up by the pelvis. Then grip his waist with your thighs	Aside from being a fabulous arm workout for you, this male-dominant sex position allows him deeper penetration	Negotiable
6	‘Leap Frog’	This is a modified doggy-style. Get on your hands and knees, then, keeping hips raised, rest your head and arms on the bed	This sex position creates deeper penetration and gives you a chance to rest on a pillow	Negotiable
7	‘Stand and Deliver’	With both of you standing, you bend over at the waist; he enters you from behind	Bending over during this sex position helps make the vaginal walls tighter and increases the intensity of the friction	Negotiable
8	‘Magic Mountain’	Your partner sits, legs bent, leaning back on his hands and forearms. You do the same and then inch toward him until you make contact	You will both feel really connected looking at each other. Increase your stimulation by grinding your clitoris against his pelvis	Negotiable
9	‘Cowgirl’	You kneel on top, pushing off your partner's chest and sliding up and down his thighs. You can relieve some of your weight from his pelvis by leaning back and supporting yourself on his thighs	By being the dominant in this sex position, you’ll delay his climax and intensify yours	Negotiable
10	‘Cowboy’	You lie on your back while your partner straddles you and then gently inserts his penis through the tight opening created by your semi-closed legs	Tightness increases the intensity of the penetration	Negotiable
11	‘Ballet Dancer’	Standing on one foot, face your partner and wrap your other leg around his waist while he helps support you	This sex position allows for quality face time and connecting	Negotiable
12	‘Scoop me up’	Both of you lie on your sides, facing the same direction. You bring your knees up slightly while your partner slides up behind your pelvis and enters you from behind	This sex position allows for more skin-to-skin contact, increasing your stimulation	Negotiable
13	‘The Chairman’	Your partner sits on the edge of the bed and you sit on him, facing away	This sex position will hit the G-spot. Meanwhile, you can use your hands to stimulate his scrotum or perineum	Negotiable
14	‘Spork’	While you lie on your back, raise your right leg so he can position himself between your legs at a 90-degree angle and enter you. Your legs will form the tines of a spork. You can do this with him facing you or facing your back	From the spork position, you can lift your top leg and support it by resting it on your partner's shoulder. From here, you can easily stimulate your clitoris using your fingers while he is inside you	Negotiable
15	‘Table Top’	You don’t have to do this one on a table. Any surface that hits your partner at crotch height will do. Have him enter you while you’re sitting or lying at the edge of a table, counter, or maybe even your bed	This position is great for face-to-face action. Plus, if you two are drastically different heights, this is a great option, since it puts you both at the same height	Negotiable
16	‘Champagne Room’	Your partner sits and you sit on top of him, facing away	It helps you regulate the pace and intensity of the thrusts	Negotiable
17	‘The Lazy Man’	Place pillows behind your partner's back and have him sit on the bed with legs outstretched. Now straddle his waist, feet on the bed. Bend your knees to lower yourself onto him, using one hand to direct his penis in. Just by pressing on the balls of your feet and releasing, you can raise and lower yourself onto his shaft as slowly or as quickly as you please	This position puts you in control, and maintains plenty of intimacy. Think of his penis as a masturbatory tool, something to rub and stimulate your clitoris with and against	Negotiable
18	‘Snow Angel’	Lie on your back and have your partner straddle you facing away. Lift your legs and wrap them around his back to elevate your pelvis so he can enter you. Grab his butt to help him slide up and back. Add a little massage action to your grip	You get a prime view of his cute butt. Plus, from this position, you have easy access to fondle his testicles. Not to mention, his pelvis is perfectly positioned to grind against your clit	Negotiable
19	‘The Snake’	Lie down on your stomach, and have your partner lie down on top of you and slide in from behind	This position allows for super-deep penetration, and a snug fit which can feel great for you and your partner	Negotiable
20	‘Blowjob’	The blowjob involves using the hands and mouth, fingers, skilled tongue and even breast: caress his penis gently, be sufficiently rude, and the result will not be long in coming	The man is entirely under your control and you may do with him everything you want	Negotiable

### The reasoning behind the prizing of commercial sex services

Explaining the reasoning behind prizing of commercial sex services some respondents indicated that:

We usually charge high prices because we are university students with a high class (Female, undergraduate student)

Another respondent indicated that:

Some sexual positions/styles are very aggressive and painful so we charge more to compensate for endurance and buy medications in case of injuries (Female, undergraduate student).

Another respondent explained:

The missionary position of sexual intercourse is the cheapest because you just have to lie on your back while the man lies face down on top of you and do the work as you relax and even be sleeping if you want to (Female, postgraduate student).

The pricing strategies by male student commercial sex workers vary from those of their female counterparts. A respondent indicated that:

For us men, we don’t have customers regularly so our pricing is dependent on the type of customer we get and the situation in which we find ourselves at the time (Male, undergraduate student).

Another male respondent explained:

We charge foreigners more than the locals. Sometimes we charge about US$100 per ejaculation without condoms and US$50 with condoms (Male, undergraduate student) (US$ 1 would be equivalent to GHS 5.8)

Another respondent was of the view that:

My main target is male foreigners although sometimes I also service some sugar mommies on call. Some of the males are homosexual colleague students and university workers who come to my room for sex. In the case of the sugar mommies, they call me on phone and we arrange a place where outside campus we meet, fuck, get my cash, and leave (Male, undergraduate student).

As to whether one is able to offer all the styles enumerated in Table 3 when required, a respondent indicated that: “––why not! Since I’m a master of my business, I always devise strategies to cope. I also learn new styles everyday on the internet, eat well, dress well to keep myself sexy and attractive to men”* (Female, diploma student).*

Another respondent was of the view that:

Apart from penetrative sex, oral stimulation of the penis, exposure of my vagina to a client and foreplays usually stimulate sexual pleasure and in most cases, some men ejaculate without penetration and have to pay for the service since ejaculation has occurred (Female, undergraduate student).

### Initiation into commercial sex work

The respondents recalled how they got initiated into the sex business. In most cases, it was peer influence on account of making money and other material gains for personal upkeep whilst in school. Whereas about 35% of the respondents were commercial sex workers before gaining admission into the universities, the majority (75%) of them including the males were introduced into the commercial sex work during the second year of admission into the university and predominantly by their room, course, social clubs and/or study mates. The sophisticated lifestyles of peers on campus coupled with the financial or material gains benefited from the peers were key motivations for easy initiation into commercial sex work.

……..I don’t really know how it started––––- but all I can say for now is, one thing led to the other and now I’m into this business to meet my financial needs whilst in school (Male, undergraduate).

I’ve been doing this thing since my secondary school days even before coming to this university. I used to work in a night club where I was thought how to manage male customers for my boss….. Later, I left that nightclub and started doing my own thing because my boss was cheating me…….. ((Female, undergraduate).

It’s a long story……My first enter with this work was when I lost my parents two years ago at level 200 and was compelled to take care of myself and two little siblings whilst in school……With no source of help, two younger siblings to fend for and myself, life was so difficult that I easily gave in when my friends introduced me to the business that I found financially sustaining for now (Female, undergraduate).

### Reasons for commercial sex work

The respondents enumerated various reasons other than financial or material gains for becoming a sex worker. Some respondents explained as follows:

I’m from a very rich home and my friends are surprised to know that I’m in this commercial sex business with them. I don’t need money from the men I sleep with, but good sex, which makes me relax, emotionally stable and puts me in a good mood to study for my exams. I not usually satisfied by one man but multiple sexual partners (Female, undergraduate).

Another respondent indicated that:

I sometimes beg and pay guys to have sex with me in my hostel. In situations when this becomes a challenge, I step onto the street with the intention not to make money but to satisfy myself by having sex with multiple men in brothels. I’m gainfully employed on study leave but not interested in any man for a long/marital relationship (Female, postgraduate student).

### Regular customers of student sex workers, meeting places, and form of remuneration

Data was also collected on the most regular patrons of student sex workers, meeting places for sexual encounters, and forms of remunerations in descending order (Table 4).

**Table 4:** Regular customers, meeting places and form of remuneration for services. *Source*: Field data 2019

Regular customers	Regular meeting place for sex	Form of remuneration
1. University workers (teaching and non-teach staff)	Hotels, guest houses, student hostels, customer’s residences and brothels	Money, sex for pleasure material and academic favors
2. University Students	Hotels, guest houses, student hostels and brothels	Money, academic support, sex for pleasure
3. Others (customers outside the university environment)	Hotels, guest houses, student hostels, customer’s residences brothels and movie houses	Money and material gains

### Challenges encountered by the student sex workers whilst in the sex business

Violence, unwanted pregnancy, and Sexually Transmitted Infections were key challenges reported by the student sex workers. A respondent indicated that:

Sometimes we are beaten up by men in their houses when they pay us and we refuse to do what they expect from us so now we seek protection from pimps (Female, undergraduate student).

Another respondent indicated that:

There are situations when we become pregnant or get some sexually transmitted diseases. This is why we charge higher if a customer wants raw sex or in the event that a condom gets bursts in action so that we can quickly buy medications to prevent pregnancy and any sexually transmitted diseases (Female, postgraduate student).

……..It’s sometimes difficult to avoid unprotected sex in this business no matter how hard we try. There are situations where some customers will start fucking you with a condom on mutual agreement, but will later remove the condom and forcefully fuck you especially when the man is on top of you.…..Other customers are able to negotiate and give additional money or even double the price to enable us accept unprotected sex……… Others also become so aggressive if we refuse them unprotected sex, hence they physically and sexually abuse us…….… (Female undergraduate student.

Respondents are very much aware of the various risks associated with commercial sex work. They have therefore derived various strategies to mitigate the business associated risks (Table 5).

**Table 5:** Protection against violence, pregnancy and sexually transmitted infections. *Source*: Field data 2019

Potential threats	Occurrence	Actions taken
Violence	Usually occurs when a client’s demand is high but refuses to pay accordingly	We always have macho men standing by as pimps who escort us secretly to such places to protect us when need be
	Usually occurs when a client refuses to pay after sex	
	Usually occurs when a client takes you home and other men comes to take their turns but refuses to pay after sex	
Unwanted Pregnancy	When condoms burst	Using regular contraceptives particularly 3 months injections to prevent pregnancy and also avoid menstruation to keep in business Uses emergency contraception after unprotected sex Have an abortion if pregnancy is established accidentally
	When a client has sex without using condoms	
Sexually transmitted infections	When a client has sex without using condoms	I select my clients wisely Only healthy looking customers are allowed to have sex with me without condoms We check their genitals to ensure there are no sores, discharge or skin rashes before sex

Some respondents also indicated that commercial sex work has some negative consequences on their academic performance in school. A respondent indicated that:

Sometimes ‘business’ becomes so good that I forget about lectures and follow my customers on appointments. When this happens, I’m not able to partake in class assignments and even miss examinations (Male, undergraduate student).

Another respondent was of the view that:

Although I pay people to write lecture notes and do assignments for me when I’m absent, it’s always difficult to understand the notes so I always don’t perform well in my final exams although I’ve not been referred in any paper (Female, diploma student).

Another respondent was of the view that:

My customers sometimes disturbs me so much that I end up losing concentration in school hence not able to attend classes regularly as expected (Female, undergraduate student).

Some of the female students have devised various strategies including offering sexual favors to some lecturers in exchange for marks to pass examinations. One of such students indicated that:

I have a way of seducing my lecturers and teaching assistants to give them good sex in exchange for good marks. The challenge however is, it’s not all of them that are morally week to succumb to my seducing no matter how hard I try. I, therefore, pay critical attention to the course being taught by such lectures and teaching assistants in order not to fail (Female, undergraduate student).

On the contrary, some respondents indicated that they are able to combine sex work and academic work effectively since they claimed to have been among the top-performing students in their class practicing commercial sex work from the undergraduate level till now at the postgraduate level.

## Discussion

In Ghana, commercial sex work is stigmatized and illegal but widespread in communities so much so that many Ghanaians are unaware that it is prohibited [21, 22]. Despite the social stigma and criminalization of commercial sex work in Ghana, the practice is becoming common among public university students. Various strategies including hanging out in drinking bars around university campuses at night to be picked, leaving contact numbers and pictures in hotels and guest houses around the universities to be contacted by prospective customers when services are required, while others operate in their hostel rooms or rented private hostels around the campuses. Brothels are also springing up in and around the various university campuses with some branded as movie houses.

The predominant background characteristics of university students observed to have been indulged in commercial sex work is a piece of evidence that the sex business on public university campuses involves young unmarried people of various nationalities who have various social and economic needs believed to be causing a proliferation in the sex business on public university campuses in Ghana. Although in the minority (4%), some male students were also found indulging in commercial sex work targeting wealthy men believed to be homosexuals and women (‘sugar mommies’) predominantly for financial gain.

The huge numbers of foreign students (81%) who indulged in commercial sex work on the various public university campuses confirm media reportage that *‘*commercial sex work *is illegal in Ghana but we have not placed strict restrictions on sex tourism in preventing HIV/AIDS, especially when sex is transacted between two consenting adults’* [23]. Consequently, commercial sex work is being practiced on the campuses of the various public universities in various forms, and students adopt various strategies and coping mechanisms for their sex business whilst in school; not only as means for economic survival but sometimes a basic need for sexual gratification to cope effectively with academic work on campus. These findings conform to those of other studies [24–26] which asserted that a large number of university undergraduates indulge in commercial sex work on various University campuses. With the associated risk of HIV and other sexually transmitted infections, a study conducted in Southeast Ethiopia shows that the youths including university students are at higher risk of contracting STIs including HIV infections due to their risky sexual behaviors [27].

In the current study, we observed that commercial sex work is becoming a common practice on the campuses of the various public universities in various forms and for various reasons as the students involved adopt various strategies and coping mechanisms for their sex business whilst in school. This observation is not only for economic survival of the students involved but sometimes a basic need for sexual gratification to cope effectively with academic work on campus. Although each of the indicated factors associated with commercial sex work in this study may encourage university students to become involved in commercial sex work, a similar study, also observed biological, psychological, environmental, cultural, and economic factors in the form of the difficult financial situation during studies, above-average sex drive, laziness, and consumer mentality supported by the culture of promiscuity, and blamed the situation more on the lack of sexual education on university campuses which creates a conducive environment for risky sexual behaviors among young people on campus [26].

Commercial sex work among students was observed as a business and the cost of service was determined by place, duration of sex, type of customer, type of services as well as sexual position/style. In most cases, the cost of patronizing the services of a commercial sex worker is negotiable. However, the perceived risk and aggressiveness of the sexual act determine the cost of service and in most cases, the money is collected before the sexual encounter begins. Special negotiable charges apply for anal sex, oral sex, and sexual intercourse without condoms as these were perceived as high-risk sexual acts.

Twenty (20) different sexual positions/styles were enumerated and priced accordingly. The Short time and missionary position of sexual intercourse was reported to be the cheapest and most patronized among the package because it’s perceived as stress-free. Explaining the reasoning behind prizing of commercial sex workers’ services, it was noted that some sexual positions/styles are very aggressive and painful hence the costs for such styles are usually high to compensate for endurance and buy medications in case of injuries. The missionary position of sexual intercourse was observed as the cheapest among the 20 enumerated sexual positions available due to its convenience and stress free nature for the female sex workers. The costs of the other styles are negotiable based on the environment for the sexual encounter, duration and perceived risk by the commercial sex workers.

The observation that customers are made to pay more for unprotected sex and in the event of condoms bursting is an indication that the students’ sex workers are aware of the health risk of unprotected sex but still indulge in it and charging more to be able to pay for the associated health outcomes.

In this study, the authors also observed that the female student sex workers in particular make more money if they agree to sex without a condom (unprotected sex). In some situations, their customers start the sexual act with a condom on a mutual agreement but later break the agreement forcefully to continue the sexual act without a condom despite the associated risks of contracting STIs and unplanned pregnancy. In some situations, some of the customers are able to lure the student sex workers with additional money to accept unprotected sex, whilst others are physically abused if they insist on protected sex. This observation, particularly among students of higher institutions of study, defeats the public health efforts of preventing new infections and spread of STIs including HIV among young people and their patrons [24]. A study on commercial sex work in Ghana indicates that the effectiveness of AIDS control programs in Ghana depends on an enhanced understanding of the social and economic dynamics of commercial sex work [22]. This is one typical situation that requires an enabling environment devoured of stigma and judgmental behaviors from the community to empower commercial sex workers who fall victims to such acts to be able to seek justice and medical care.

### Initiation into commercial sex work

The act of initiation into commercial sex work and reasons for becoming a student commercial sex worker in a public university in Ghana, as noted in this study is multifaceted. Peer influence on account of making money and other material gains for personal upkeep was a key determinant for commercial sex work as observed in this study. Some studies have also linked negative peer influences on commercial sex work [27, 28]. The university environment for some undergraduate students is a transitionary phase, filled with mixed feelings of excitement, new experiences, autonomy, stress, and loneliness, since some students have to navigate between academic work and social demands [29]. With this transitionary phase, affluent and well-exposed peers become the immediate associates and sometimes mentors to the less affluent hence exposes their less exposed colleagues to various survival strategies, including how to acquire money and material things whilst in school [30]. These peer influences sometimes become unhealthy thereby resulting in student sex workers for financial/materials gains [31, 32].

On the contrary, the current study found that commercial sex work among students in public universities in Ghana is not only for financial/materials gain but also for emotional stability as revealed that some students became sex workers because in their view they become more emotionally stable, relaxed and perform academically better if they have sex with multiple partners prior to examinations. Although this could be seen as sex addiction, such students think such an addiction is normal [33]. Relating the practice to Maslow’s hierarchy of needs [34], the authors are compelled to justify this behavior as a means to an end if that could help such students achieve their needs on campus. Within the Ghanaian context, a perception with such behavior however could be attributable to demonic possessions that would require deliverance [35].

Commercial sex work among students is associated with various risks such as violence, unwanted pregnancy, and STIs. Various strategies such as paid protection and extra charges to mitigate these business associated risks are adopted. With regard to effects on academic performance, the general observation has been that there are various consequences of students indulging in commercial sex work on their academic performance. The opinion of the University students as observed in a previous study [36] that the impact of commercial sex work on their academic performance is that they do not usually pass their examination as expected and therefore do not graduate on time was also noted in the current study. More than half of the student sex workers had at least two referral papers to write at every level of their academic life.

Consequently, some of the female students reported offering sexual favors to some lectures in exchange for marks to pass exams. The presence of male student sex workers on university campuses and their reasons for indulging in the sex work in school supports findings of another study on male sex workers which observed that such males resorted to commercial sex work when they are desperately short of money to the extent of having no access to food or shelter [37]. This practice is very unusual for the expectations of men within the Ghanaian culture and explains the negative influence of some foreign cultures on Ghanaian university students. Whilst good upbringing is generally perceived as a remedy for indulging in commercial sex work [38, 39], this study has shown that commercial sex work among university students goes beyond ‘good up bring’ since there is more to it than only upbringing or good parenting.

## Conclusion

The results of this study are revealing as they show that students from the major public universities in Ghana indulge in commercial sex work in and around the various university campuses for financial, material, and emotional gains. The finding that student sex workers have devised various strategies including offering sex for marks as a way to combine academic work and the sex business requires further investigations to explore its extent. The springing up of brothels and movie houses in and around the university campuses and the practice of students’ converting their hostel rooms into brothels requires law enforcement to prevent or regulate such practices in an academic environment. Price negotiation based on the environment, duration, and styles/positions adopted for sex does not only expose the level of commercializing sex work amongst students in public Universities in Ghana but also the conscious and unconscious socio-economic, cultural, behavioral and moral factors that predispose students to commercial sex work at the university. The Ministry of Education, University authorities, and Non-Governmental organizations (NGOs) are required to develop programs on sexuality education to ensure safer sex practices as a human rights issue in the universities to prevent the spread of sexually transmitted diseases including HIV/AIDS among students. There is a need for further studies in this area and a multi-sectoral approach for appropriate policy and program interventions to regulate the practice on university campuses in Ghana.

### Limitations of the study

Funding was a major limitation to the study as the authors self-fund the study. This financial constraint affected the duration of the fieldwork for extensive data collection. In some cases, the inability of the authors to pay huge sums of money (GHS 500 per hour interview) demanded particularly by some of the perceived ‘high class’ student sex workers as compensation for their time lead to their refusal to grant any interviews or pulled out midway the interviews. We advise that adequate funding to pay for respondents’ time should be solicited for future studies of this kind in Ghana.

## Supplementary Information

**Additional file 1.** Questionnaire for student sex workers.

**Additional file 2.** In-depth interview guide for key informants.

**Additional file 3.** In-depth interview guide for student sex workers.

## Data Availability

The raw data collected is available upon reasonable request from the corresponding author.

## Abbreviations

AIDS: Acquire Immune Deficiency Syndrome
CSW: Commercial Sex Work
GHS: Ghanaian cedi
HIV: Human Immune Virus
IDIs: In-Depth Interviews
SPSS: Statistical Package for Social Sciences
STIs: Sexually Transmitted infections
US$: United States Dollars
