# Supplementary material for: Commercial sex work among university students: a case study of four public universities in Ghana
Source: BMC Womens Health. 2021 Mar 10;21:103. doi: 10.1186/s12905-021-01251-2 (PMC7971108; doi:10.1186/s12905-021-01251-2)
Supplement: Supplementary file 1 — Additional file 1. Questionnaire for student sex workers. [file 12905_2021_1251_MOESM1_ESM.docx]

**UNIVERSITY OF EDUCATION, WINNEBA**

**QUESTIONNAIRE**

**(Commercial sex work among university students: A case study of four public universities in Ghana)**

**DATA PROCESSING PARTICULARS**

Place where respondent was interviewed _____________________________

Medium of communication during the interview__________________________

Interview Date:_____________________________________________________

***A).* BACKGROUND CHARACTERISTICS OF RESPONDENTS**

*[I would like to ask you a few questions regarding your background. Please feel free to answer me]*

Background Characteristics of respondents

| **Age in years** | | |
| --- | --- | --- |
| <18 | [ ] |  |
| 18- 24 | [ ] |  |
| 25-31 | [ ] |  |
| >31 | [ ] |  |
| **Religion** | | |
| Christian | [ ] |  |
| Muslim | [ ] |  |
| Other specify------------------------------- | | |
| **Sex** |  |  |
| Males | [ ] |  |
| Females | [ ] |  |
| Other specify----------------------- | | |
| **Marital status** | | |
| Married | [ ] |  |
| unmarried | [ ] |  |
| Other specify----------------------- | | |
| **Nationality** | | |
| Ghanaian | [ ] |  |
| Nigerian | [ ] |  |
| Liberian | [ ] |  |
| Other specify------------------------------ | | |
| **Primary care taker of respondents** | | |
| Parents | [ ] |  |
| Siblings | [ ] |  |
| Partner | [ ] |  |
| Self | [ ] |  |
| Other specify------------------------------- | | |
| **Student residential status** | | |
| On campus university hostel | [ ] |  |
| On campus rented private hostel | [ ] |  |
| Off campus rented apartments | [ ] |  |
| Other specify----------------------------------------------- | | |
| **Institution of study-------------------------------------** | | |
| **Program of study** | | |
| Humanities | [ ] |  |
| Social Sciences | [ ] |  |
| Health Sciences | [ ] |  |
| Pure Sciences | [ ] |  |
| Other specify------------------------------ | | |
| **Level of study** |  |  |
| Certificate | [ ] |  |
| Diploma | [ ] |  |
| Undergraduate | [ ] |  |
| Postgraduate | [ ] |  |
| Other specify----------------------------- | | |
