# Supplementary material for: Commercial sex work among university students: a case study of four public universities in Ghana
Source: BMC Womens Health. 2021 Mar 10;21:103. doi: 10.1186/s12905-021-01251-2 (PMC7971108; doi:10.1186/s12905-021-01251-2)
Supplement: Supplementary file 2 — Additional file 2. In-depth interview guide for key informants. [file 12905_2021_1251_MOESM2_ESM.docx]

**UNIVERSITY OF EDUCATION WINNEBA**

**IN-DEPTH INTERVIEW GUIDE FOR KEY INFORMANTS**

**(Commercial sex work among university students: A case study of four public universities in Ghana)**

**DATA PROCESSING PARTICULARS**

Place where respondent was interviewed _____________________________

Medium of communication during the interview__________________________

Interview Date: ____________________________________________________

[I will like to ask you some questions about your business. Some of these questions may be quite sensitive, and might make you uncomfortable, but be as frank as possible. I will like to assure you that your responses will be kept confidential.]

1. ***Background Information***

- Please tell me about yourself and your work here

**Probe:** type of work, what it entails, duty post/office location, roles/responsibilities, etc.

1. **Experiences with commercial sex workers on university campuses**

- Please are there commercial sex workers on/around this university campus?

**Probe:** Students’ involvement, locations, clienteles, patronage, level of involvement/support, etc.

1. **The emergence of commercial sex work on university campuses**

- Please tell me when and how commercial sex work started on this campus.

**Probe:** how it started, where it started, those involved, how it’s practiced, law enforcement, etc.

1. **Challenges encountered with the student sex workers**

- Please tell me about some challenges you have ever encountered with the student sex workers

**Probe:** Situation, Behaviour, Impact, etc.
