# Supplementary material for: Commercial sex work among university students: a case study of four public universities in Ghana
Source: BMC Womens Health. 2021 Mar 10;21:103. doi: 10.1186/s12905-021-01251-2 (PMC7971108; doi:10.1186/s12905-021-01251-2)
Supplement: Supplementary file 3 — Additional file 3. In-depth interview guide for student sex workers. [file 12905_2021_1251_MOESM3_ESM.docx]

**UNIVERSITY OF EDUCATION WINNEBA**

**IN-DEPTH INTERVIEW GUIDE FOR STUDENT SEX WORKERS**

**(Commercial sex work among university students: A case study of four public universities in Ghana)**

**DATA PROCESSING PARTICULARS**

Place where respondent was interviewed _____________________________

Medium of communication during the interview__________________________

Interview Date:_____________________________________________________

[I will like to ask you some questions about your business. Some of these questions may be quite sensitive, and might make you uncomfortable, but be as frank as possible. I will like to assure you that your responses will be kept confidential.]

1. **Background Information**

- Please tell me about yourself?

**Probe:** Age, religion, place of residence, marital status, number of living children, level of education at the University, sponsor(s), etc.

1. **The emergence of commercial sex work on university campuses**

- Please tell me when and how commercial sex work started on this campus.

**Probe:** how it started, where it started, those involved, how it’s practiced, law enforcement, etc.

1. **Mode of operations (modus operandi)**

- Please tell me how you go about your work here

**Probe:** how the sex workers go about their work on the campuses, available supports, means of getting clients, where they go with their clients to have sex, cost of services, etc.

1. **Respondents’ view about commercial sex work**

- Please tell me your views about commercial sex work.

**Probe:** perceptions about sex work, regrets, motivators, perceived risk, etc.

1. **Determinants of cost of commercial sex workers’ services**

- Please tell me how much are your services

**Probe:** Cost of services, the reasoning behind the pricing of services, etc.

1. **Initiation into commercial sex work**

- If you can recall, at what age did you start prostitution? When did you start prostitution? What made you start commercial sex work?

**Probe:** Circumstances leading to the initiation into commercial sex work, motivators and reasons to continue sex work.

1. **Regular customers of student sex workers, meeting places and form of remuneration**

- Please tell me some of your regular customers, where you meet them and what you get from them.

**Probe:** Regular customers, meeting places for sex, the form of remuneration (cash, kind, or both).

1. **Challenges encountered by the student sex workers in the sex business**

- Please tell me some of the challenges you encounter combining this work with your studies

**Probe:** Violence, abuse, academic setbacks, ill health, injuries, fatigue, stigma, etc
